# Metformin suppresses triple-negative breast cancer stem cells by targeting KLF5 for degradation

**DOI:** 10.1038/celldisc.2017.10

**Published:** 2017-04-18

**Authors:** Peiguo Shi, Wenjing Liu, Tala, Haixia Wang, Fubing Li, Hailin Zhang, Yingying Wu, Yanjie Kong, Zhongmei Zhou, Chunyan Wang, Wenlin Chen, Rong Liu, Ceshi Chen

**Affiliations:** 1 Key Laboratory of Animal Models and Human Disease Mechanisms of the Chinese Academy of Sciences and Yunnan Province, Kunming Institute of Zoology, Chinese Academy of Sciences, Kunming, China; 2 Kunming College of Life Science, University of Chinese Academy of Sciences, Kunming, China; 3 Faculty of Life Science and Technology, Kunming University of Science and Technology, Kunming, China; 4 State Key Laboratory of Medicinal Chemical Biology, College of Life Sciences, Nankai University, Tianjin, China; 5 School of Life Sciences and Medical Center, University of Science and Technology of China, Hefei, China; 6 Department of Pathology, First Affiliated Hospital of Kunming Medical University, Kunming, China; 7 Cancer Hospital, Kunming Medical University, Kunming, China

**Keywords:** KLF5, metformin, PKA, stem cells, triple-negative breast cancer

## Abstract

Out of the breast cancer subtypes, triple-negative breast cancer (TNBC) has the poorest prognosis without effective targeted therapies. Metformin, a first-line drug for type 2 diabetes mellitus, was demonstrated to target breast cancer stem cells selectively. However, the efficiency and the mechanism of action of metformin in TNBC are unclear. In this study, we demonstrated that metformin decreased the percentage of TNBC stem cells partially through the downregulation of the expression of the stem cell transcription factor Krüppel-like factor 5 (KLF5) and its downstream target genes, such as *Nanog* and *FGF-BP1*, in TNBC cell lines. Metformin induced glycogen synthase kinase-3β (GSK3β)-mediated KLF5 protein phosphorylation and degradation through the inhibition of protein kinase A (PKA) activity in TNBC cells. Consistently, PKA activators increased the expression levels of KLF5. We observed a positive correlation between p-CREB, p-GSK3β, KLF5 and FGF-BP1 protein levels in human TNBC samples. These findings suggest that metformin suppresses TNBC stem cells partially through the PKA-GSK3β-KLF5 signaling pathway.

## Introduction

Breast cancer is the most common cancer in women in China and the United States. China was estimated to have 268 600 new cases in 2015, and the United States was estimated to have 246 660 new cases in 2016 [1, 2]. Triple-negative breast cancer (TNBC; estrogen receptor α-, progesterone receptor and HER2-negative) is the subtype of breast cancer with the poorest prognosis due to a lack of targeted therapies [3]. TNBC can further be divided into different subtypes, including the basal-like and claudin-low subtypes [4]. Basal-like TNBC accounts for ~80% of TNBC [4]. Breast cancer stem cells, also known as breast tumor-initiating cells, are responsible for cancer metastasis [5–7], chemoresistance [8] and recurrence [9]. Therefore, a good strategy might be to target TNBC stem cells to treat TNBC.

Human Krüppel-like factor 5 (KLF5, also named BTEB2 and IKLF) is a member of the KLF family [10]. KLF5 is regulated by multiple signaling pathways, including those mediated by Hippo [11], Wnt [12], Notch [13] and Ras [14]. Recently, Joan Massagué and his co-workers [15] reported that KLF5 has a key role in TGF-β-induced tumorigenesis of pancreatic ductal adenocarcinoma. Accumulating evidence suggests that KLF5 is a potential therapeutic target for TNBC. Our previous studies suggest that KLF5 promotes TNBC cell proliferation [16], survival [17], migration and invasion [18], as well as stemness [19]. We reported that mifepristone suppresses TNBC stem cells through the downregulation of KLF5 [19].

Metformin is a first-line drug used for type 2 diabetes mellitus. Remarkably, metformin was reported to reduce cancer incidence [20]. Metformin also improved the prognosis of cancers, such as liver cancer, ovarian cancer, colorectal cancer, pancreatic cancer and breast cancer [21]. Breast cancer patients who were treated with metformin had significantly higher survival rates than patients who did not receive metformin [21]. Metformin selectively targets breast cancer stem cells, significantly reduces breast tumor growth and prolongs remission when combined with chemotherapy [22]. TNBC cell lines are more sensitive to metformin than non-TNBC cell lines in terms of apoptosis [23]. In claudin-low TNBC cell lines, metformin induces apoptosis and inhibits mammosphere formation through the induction of miR-193b [24] and inhibits TGF-β-induced proliferation, motility and invasion [25]. However, the mechanism of action of metformin in basal-like TNBC stem cells is unknown. Furthermore, metformin use during adjuvant chemotherapy does not significantly impact survival outcomes in diabetic patients with TNBC, although it appears to decrease the risk of distant metastasis [26]. Therefore, whether metformin is effective in cases of TNBC deserves further investigation.

In this study, we demonstrated that metformin decreases the percentage of stem cells in TNBC-derived cell lines and that metformin targets the KLF5 protein for degradation in TNBC cells. The mechanism involves the inhibition of protein kinase A (PKA) activity, which is a protein that phosphorylates glycogen synthase kinase-3β (GSK3β). GSK3β is a well-known protein that phosphorylates KLF5 at S303, which leads to KLF5 ubiquitination and degradation. Our findings suggest that metformin might be an effective therapeutic drug for patients with TNBC.

## Results

### Metformin suppresses TNBC stem cells

**Figure 1:**
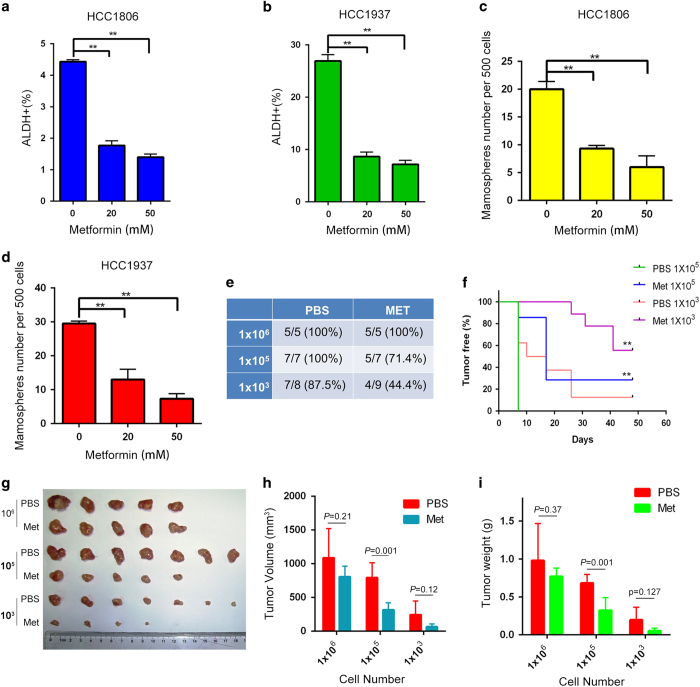
Metformin suppresses TNBC stem cells *in vitro* and *in vivo*. (**a**) In HCC1806 cells, metformin inhibited cancer stem cells (CSCs) *in vitro* according to the ALDH assay. The cells were treated with different concentrations of metformin for 24 h and digested. Twenty thousand cells were resuspended in 1 ml assay buffer for the ALDH assay. All samples were incubated for 45 min. The cells were resuspended in 0.5 ml assay buffer and subjected to flow cytometry analysis. The results are shown as bar graphs with the mean±s.d., *n*=3 independent experiments. The statistical significance was determined by Student’s *t*-test. **P<0.01. (**b**) In HCC1937 cells, metformin inhibited CSCs *in vitro* according to the ALDH assay. (**c**) In HCC1806 cells, metformin inhibited CSCs *in vitro* according to the mammosphere culture. The cells were treated with metformin for 24 h before they were plated onto 24-well ultra-low attachment plates (500 cells each well). The first passage spheres whose diameters were >60 μm after 14 days in culture were counted. The results are shown as bar graphs with the mean±s.d., *n*=3 independent experiments. The statistical significance was determined by Student’s *t*-test. **P<0.01. (**d**) Metformin inhibits HCC1937 CSCs *in vitro* as measured by the mammosphere culture. The first passage spheres whose diameters were >60 μm after 14 days in culture were counted. The results are shown as bar graphs with the mean±s.d., *n*=3 independent experiments. The statistical significance was determined by Student’s *t*-test. **P<0.01. (**e**) Metformin inhibited CSCs *in vivo* according to the limited dilution assay. HCC1806 cells were treated with metformin (40 mM) or PBS for 48 h. Cells were injected into the fat pads of 7–8-week-old BALB/C nude mice. Tumorigenesis was recorded after 7 days. The incidence of tumors in nude mice is listed based on the different cell numbers that were seeded. (**f**) The tumor-free survival curves of the nude mice that were inoculated with different numbers of HCC1806 cells with and without metformin treatment are shown. The statistical significance was determined by log-rank test. The 1×10^5^ and 1×10^3^ groups are shown. **P<0.01. (**g**) The tumors harvested at the end of the experiment. (**h**) The tumor volumes at the end of the experiment. Tumor size was measured by vernier calipers, and tumor volumes were calculated according following formula: tumor volume (mm^3^)=π(length×width^2^)/6. The statistical significance was determined by Student’s *t*-test. The difference between metformin and PBS treatment is significant (*P*<0.01) when 1×10^5^ cells were seeded. Similar trends were observed in the other groups. (**i**) Tumor weights at the end of the experiment. The statistical significance was determined by Student’s *t*-test. The difference between metformin and PBS treatment is significant (*P*<0.01) when 1×10^5^ cells were seeded. Similar trends were observed in the other groups.

To test whether metformin decreases the percentage of TNBC stem cells in TNBC-derived cell lines, we assessed the expression of the breast cancer stem cell biomarker aldehyde dehydrogenase (ALDH) [27]. Metformin decreases the ratio of cancer stem cells in the TNBC cell lines HCC1806 and HCC1937 (Figure 1a and b and Supplementary Figure S1A–C). We further confirmed this result in a mammosphere formation assay, which showed that metformin also significantly decreased the number of mammospheres formed from both cell lines (Figure 1c and d and Supplementary Figure S1D).

The golden standard for cancer stem cell testing is the limited dilution and tumorigenesis assay *in vivo*. As shown in Figure 1e, for both the vehicle control group and the metformin treatment group, 1×10^6^ HCC1806 cells formed tumor xenografts in nude mice with 100% efficiency. When 1×10^5^ HCC1806 cells were seeded, the tumor formation ratio for the control group was not decreased, but the efficiency in the metformin treatment group decreased to 71.4%. When we seeded 1 000 cells, tumors still formed in the control group with 87.5% efficiency. In sharp contrast, metformin decreased the efficiency to 44.4%. Additionally, metformin significantly extended the tumor-free survival of mice with tumor xenografts (Figure 1f) and decreased the tumor volumes and weights (Figure 1g-i).

### Metformin suppresses TNBC stem cells partially through the inhibition of KLF5

**Figure 2:**
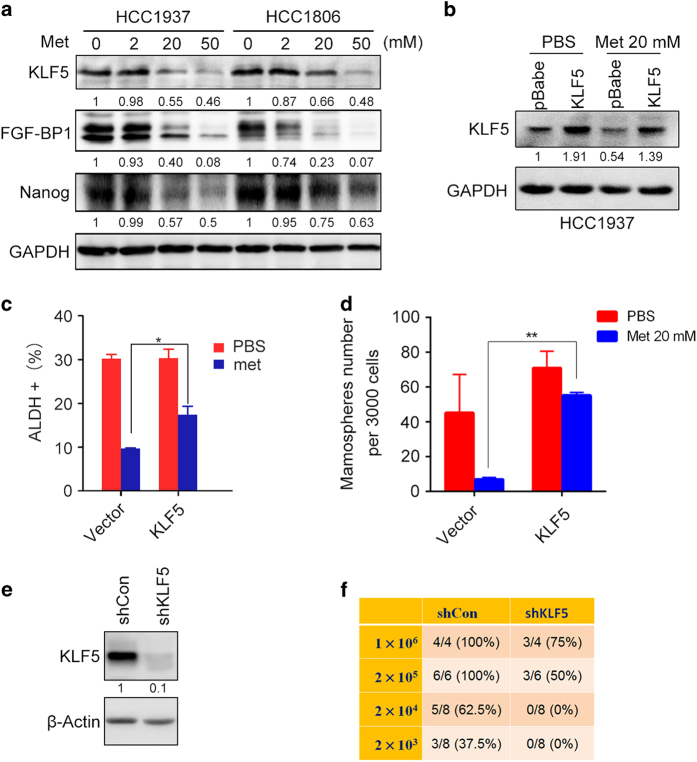
Metformin suppresses TNBC stem cells partially through the downregulation of KLF5 expression. (**a**) Metformin inhibited the expression of KLF5 and its downstream target genes, *FGF-BP1* and *Nanog*, in both HCC1937 and HCC1806 cells. Protein expression was examined by WB analysis. The cells were treated with 2, 20 or 50 mM metformin for 24 h. Glyceraldehyde 3-phosphate dehydrogenase (GAPDH) was used as the loading control. Normalized band densities were shown below each band. No metformin treatment was defined as 1. (**b**) pBabe-KLF5 or vector (pBabe) plasmids were transfected into HCC1937 cells for 48 h. The cells were then treated with either metformin (20 mM) or PBS for another 24 h, and the expression of KLF5 was detected by using the WB. (**c**) KLF5 overexpression partially but significantly rescued the metformin-induced CSC reduction in HCC1937 cells as measured by the ALDH assay. The statistical significance was determined by Student’s *t*-test*.* *P<0.05. (**d**) KLF5 overexpression significantly rescued metformin-induced CSC reduction in HCC1937 cells as measured by the mammosphere assay. The statistical significance was determined by Student’s *t*-test*.* **P<0.01. (**e**) Stable knockdown of KLF5 in HCC1806. The expression of KLF5 was tested by WB. β-Actin was used as the loading control. (**f**) Knockdown of KLF5 inhibited CSCs according to the limited dilution assay. Stable knockdown of KLF5 cells (shKLF5) and control cells (shCon) were seeded into nude mice. The incidence of tumors is listed according to cell numbers that were seeded.

KLF5 is an important transcription factor for basal-type breast cancer stem cells [19, 28]. By western blotting (WB), we found that metformin (20–50 mM) markedly downregulated the expression of KLF5 and its downstream target genes, such as *FGF-BP1* [16] and *Nanog* [29], in both HCC1937 and HCC1806 cell lines (Figure 2a). Nanog is well recognized to have a critical role in the maintenance of stemness [30]. When we overexpressed KLF5 in HCC1937 cells, the metformin-induced cancer stem cell decrease was partially rescued (Figure 2b–d and Supplementary Figure S2). We further used HCC1806 to construct KLF5 stable knockdown cells (Figure 2e), and performed the limited dilution assay to test the tumorigenesis. For 1×10^6^ and 2×10^5^ groups, the tumorigenesis of the control groups was 100%, but the tumorigenesis of KLF5-knockdown groups was 75% and 50%, respectively. For 2×10^4^ and 2×10^3^ groups, the tumorigenesis of the control groups was 62.5% and 37.5%, respectively, but the KLF5-knockdown HCC1806 cells did not form tumors (Figure 2f). These results suggest that metformin decreases TNBC stem cells partially through the inhibition of KLF5.

### Metformin decreases the stability of KLF5 protein

**Figure 3:**
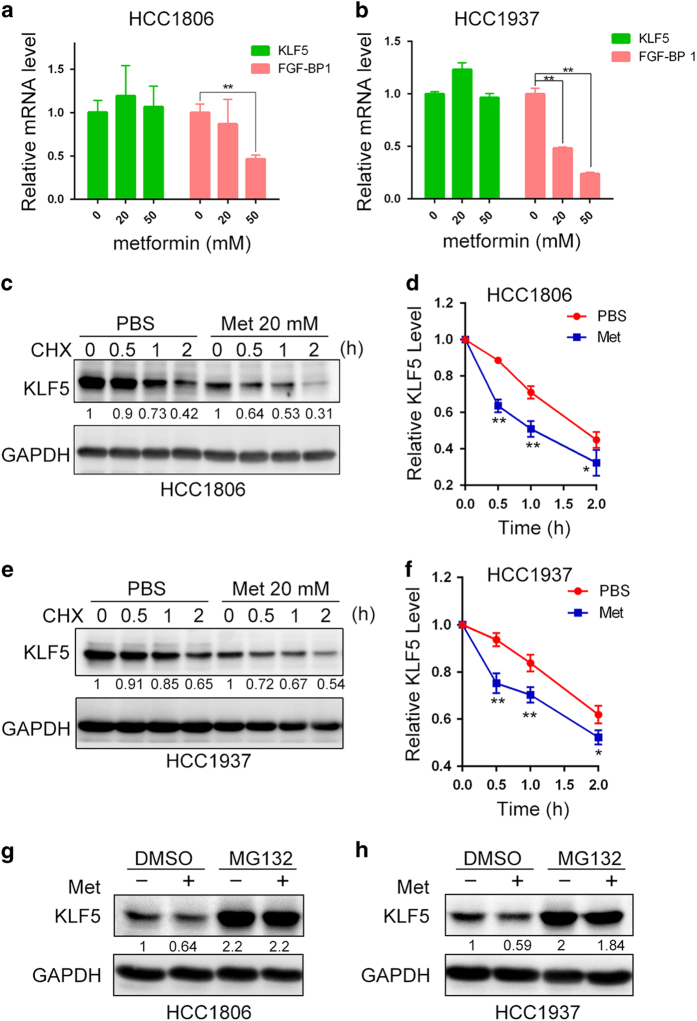
Metformin decreases the stability of the KLF5 protein. (**a**) Metformin did not decrease the mRNA levels of *KLF5* in HCC1806 cells. In contrast, metformin decreased the mRNA levels of *FGF-BP1*. The cells were treated with metformin (20, 50 mM) or PBS for 24 h and the mRNA levels were determined by quantitative RT-PCR. The statistical significance was determined by Student’s *t*-test*.* **P<0.01. (**b**) Metformin decreased *FGF-BP1* but not *KLF5* mRNA levels in HCC1937. The cells were treated with metformin (20, 50 mM) or PBS for 24 h and the mRNA levels were determined by quantitative RT-PCR. The statistical significance was determined by Student’s *t*-test*.* **P<0.01. (**c**) Metformin promoted the degradation of the KLF5 protein in HCC1806 cells. HCC1806 cells were treated with metformin (20 mM) or PBS for 24 h. Then, the cells were treated with cycloheximide (CHX, 100 μg ml^−1^) for 0.5, 1 or 2 h. The cell lysates were collected for WB. (**d**) The statistical results from panel **c**. The results are shown as bar graphs with the mean±s.d., *n*=3 independent experiments. The statistical significance was determined by Student’s *t*-test*.* *P<0.05 and **P<0.01. (**e**) Metformin promoted the degradation of the KLF5 protein in HCC1937 cells. (**f**) The statistical results from panel e. The results are shown as bar graphs with the mean±s.d., *n*=3 independent experiments. The statistical significance was determined by Student’s *t*-test*.* *P<0.05 and **P<0.01. (**g**) The proteasome inhibitor MG132 blocked the metformin-induced KLF5 decrease in HCC1806 cells. MG132 (20 μm) was used to pre-treat HCC1806 cells for 1 h. Then, the cells were treated with metformin (20 mM) for another 12 h. The cell lysates were collected for WB. (**h**) MG132 blocked the metformin-induced KLF5 decrease in HCC1937 cells. MG132 (20 μm) was used to pre-treat HCC1937 cells for 1 h. Then, the cells were treated with metformin (20 mM) for another 12 h. The cell lysates were collected for WB.

To characterize the mechanism by which metformin decreases the expression of KLF5 protein in TNBC cells, we first examined the *KLF5* mRNA levels. In both HCC1806 and HCC1937 cell lines, metformin did not decrease the *KLF5* mRNA levels, although metformin significantly decreased the mRNA levels of the KLF5 target gene *FGF-BP1* (Figure 3a and b). Then, we used a cycloheximide chase experiment to measure the half-life of the KLF5 protein. Metformin (20 mM) significantly accelerated the degradation of KLF5 in both HCC1806 and HCC1937 compared with the negative control group (Figure 3c–f). We further found that MG132, a proteasome inhibitor, blocked the metformin-induced KLF5 decrease in both cell lines (Figure 3g and h).

### Metformin downregulates the expression of KLF5 through the inhibition of PKA

**Figure 4:**
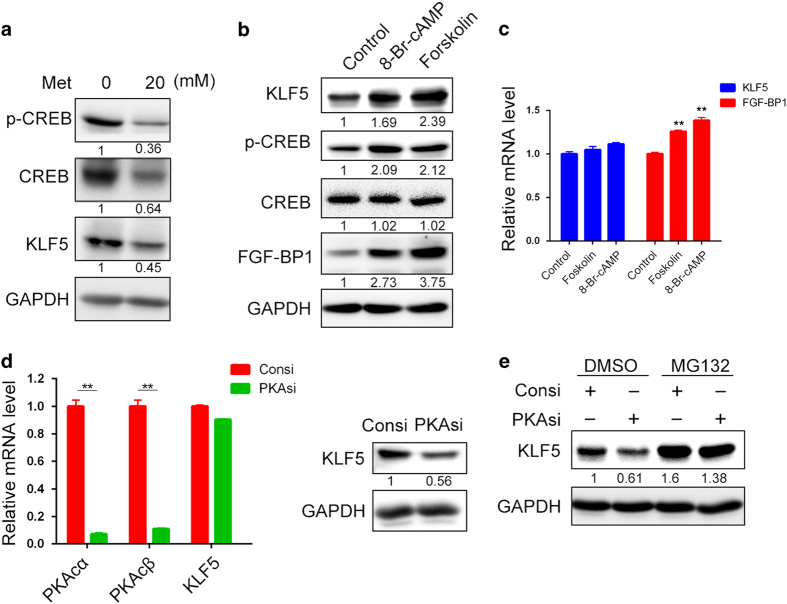
Metformin decreases the KLF5 expression through the inhibition of PKA. (**a**) Metformin inhibited the activity of PKA. HCC1937 cells were treated with metformin (20 mM) for 24 h. The p-CREB and KLF5 levels were then measured by WB. (**b**) Two activators of PKA, 8-Br-cAMP (1 mM) and forskolin (10 μm), increased the p-CREB and KLF5 protein levels, respectively. HCC1937 cells were treated with these PKA activators for 24 h. KLF5, p-CREB, CREB and FGF-BP1 were examined by WB. (**c**) Two activators of PKA, 8-Br-cAMP (1 mM) and forskolin (10 μm), did not cause an increase in the mRNA levels of *KLF5* in HCC1937 cells. However, the *FGF-BP1* mRNA level was significantly upregulated, as measured by quantitative RT-PCR. The statistical significance was determined by Student’s *t*-test*.* **P<0.01. (**d**) PKA knockdown decreased the KLF5 protein level in HCC1937 cells. PKAc was knocked down using two different siRNAs that each targeted the PKAcα and PKAcβ isoforms. HCC1937 cells were transfected with the siRNAs for 72 h, and the cell lysates were collected for qRT-PCR and WB. The statistical significance was determined by Student’s *t*-test. **P<0.01. (**e**) MG132 blocked the decrease in KLF5 induced by PKA knockdown in HCC1937 cells. The cells were transfected with PKA siRNAs for 72 h and were then treated with MG132 (20 μm) for 12 h.

We further characterized the mechanism by which metformin decreases the stability of the KLF5 protein in TNBC cells. It is well known that metformin can cause the accumulation of AMP, which inhibits the production of cyclic AMP (cAMP) and the activity of PKA [31, 32]. Indeed, metformin inhibited the PKA activity and the KLF5 protein level in HCC1937 cells. We measured the PKA activity by examining the phosphorylation level of CREB, which is a classical downstream substrate of PKA (Figure 4a). Consistently, two PKA activators, forskolin (10 μM) and 8-Br-cAMP (1 mM), markedly increased the KLF5 protein levels (Figure 4b). We further demonstrated that neither forskolin nor 8-Br-cAMP increased the *KLF5* mRNA levels. In contrast, they upregulated the mRNA level of *FGF-BP1* (Figure 4c).

To further test the hypothesis that PKA positively regulates KLF5 protein stability, we knocked down the PKA catalytic (PKAc) subunit using small interfering RNA (siRNA) and found that the *KLF5* mRNA level was not decreased (Figure 4d). However, the KLF5 protein level was downregulated (Figure 4d). MG132 also blocked the KLF5 downregulation, which was induced by the knockdown of PKA (Figure 4e).

### Metformin and PKA regulate KLF5 protein through GSK3β

**Figure 5:**
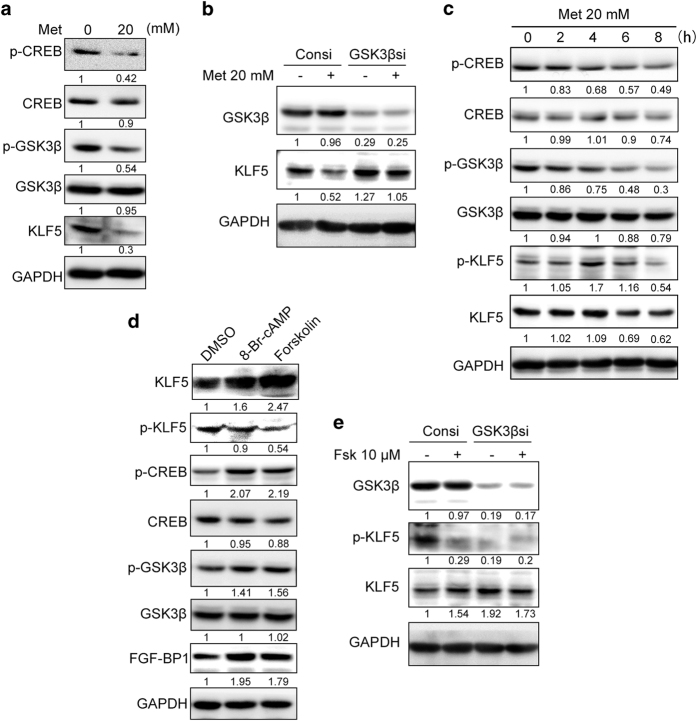
Metformin and PKA regulate the KLF5 protein level through GSK3β. (**a**) Metformin decreased the phosphorylation of GSK3β at S9. HCC1937 cells were treated with metformin (20 mM) for 24 h. The protein levels of p-GSK3β (S9), p-CREB and KLF5 were measured by WB. (**b**) GSK3β knockdown blocked the metformin-induced decrease in KLF5 expression. HCC1937 cells were transfected with either GSK3βsi or control siRNA (Consi) for 48 h. Then, the cells were treated with metformin (20 mM) for an additional 24 h. (**c**) Metformin (20 mM) decreased the protein levels of p-CREB (S133), p-GSK3β (S9) and KLF5 in HCC1937 cells in a time-dependent manner. HCC1937 was treated by 20 mM metformin for 0, 2, 4, 6 and 8 h. The cells were collected for WB to detect the p-CREB (S133), p-GSK3β (S9), p-KLF5 (S303) and KLF5 expression. (**d**) HCC1937 cells were treated with either 8-Br-cAMP (1 mM) or forskolin (10 μm), which are PKA activators, and both increased p-GSK3β (S9) and decreased the p-KLF5 (S303) protein levels. The treatment duration was 24 h. The cells were collected for WB to detect the p-CREB (S133), p-GSK3β (S9), p-KLF5 (S303), KLF5 and FGF-BP1 expression. (**e**) GSK3β knockdown blocked the forskolin (10 μm)-induced KLF5 increase in HCC1937 cells. The cells were transfected with GSK3βsi or Consi for 48 h, which was followed by the addition of forskolin for another 24 h. The cells were collected for WB to detect the GSK3β, p-KLF5 (S303) and KLF5 expression.

PKA has been shown to phosphorylate GSK3β at S9, which inactivates GSK3β [33, 34]. We previously reported that GSK3β directly phosphorylates KLF5 at the S303 site, after which the phosphorylated KLF5 protein is ubiquitinated by the SCF^Fbw7^ E3 ubiquitin ligase and degraded by the 26S proteasome [35]. To test whether GSK3β mediates metformin-induced KLF5 protein degradation in TNBC, we examined the p-GSK3β (S9) levels when HCC1937 cells were treated with metformin. Indeed, metformin (20 μM) decreased the p-GSK3β (S9) level, which was consistent with the p-CREB (S133) level (Figure 5a). When GSK3β was depleted, metformin failed to downregulate KLF5 (Figure 5b). The knockdown of GSK3β itself increased the KLF5 protein level (Figure 5b), which was in agreement with our previous results [35].

Since GSK3β directly phosphorylates KLF5 at S303 and then induces its degradation, we speculated whether metformin increases the phosphorylation of KLF5. As expected, metformin decreased the activity of PKA (p-CREB) and increased the activity of GSK3β (p-GSK3β) at 2 h following treatment. In addition, metformin increased the p-KLF5 (S303) level at 4 h after treatment. Subsequently, the level of total KLF5 protein was decreased at 6 h (Figure 5c). Additionally, PKA activators increased the p-GSK3β and decreased the p-KLF5 (S303) levels in HCC1937 cells (Figure 5d). When GSK3β was depleted, forskolin could no longer increase the KLF5 protein level (Figure 5e). Consistently, the knockdown of GSK3β also downregulated the p-KLF5 (S303) levels (Figure 5e).

### PKA activity, p-GSK3β and FGF-BP1 positively correlate with the KLF5 expression level in human TNBC

**Figure 6:**
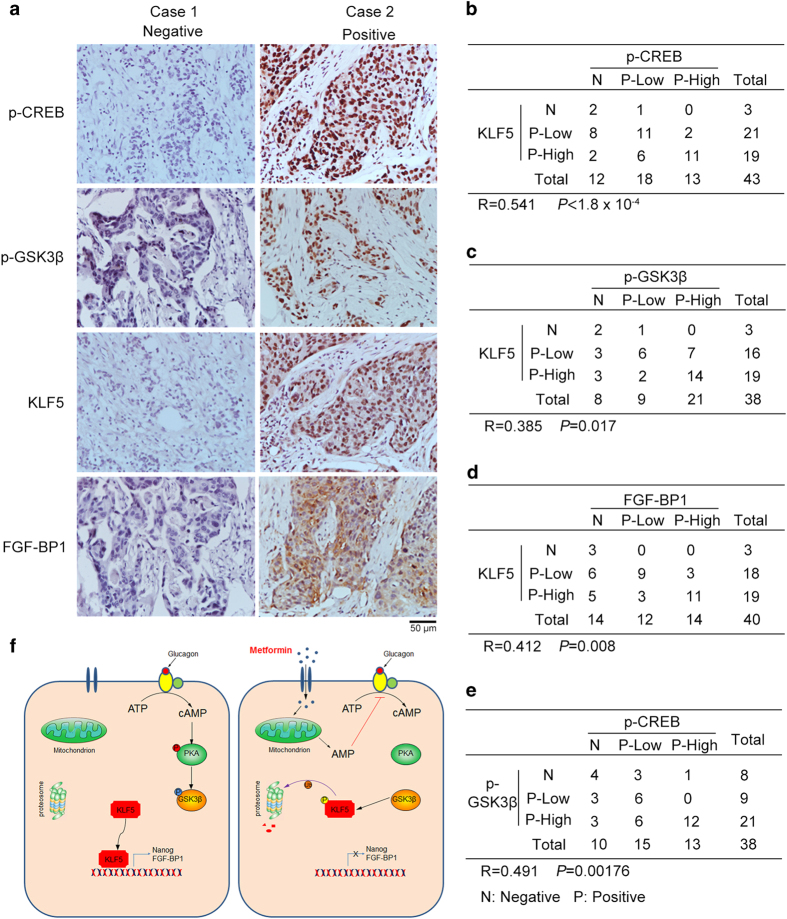
The PKA activity, p-GSK3β and FGF-BP1 positively correlate with the KLF5 expression level in human TNBC specimens. (**a**) Representative IHC results for p-CREB (S133), p-GSK3β (S9), KLF5 and FGF-BP1 in TNBC specimens. Tumor cells that did not stain positive were defined as 'negative'. Positive cells that accounted for <50% of cells were defined as 'Positive-Low'. Positive cells that accounted for >50% of cells were defined as 'Positive-High'. (**b**) A positive correlation was observed between p-CREB (S133) and KLF5 expression levels in the TNBC samples. Statistical significance was determined by the Spearman's test. *R* is the correlation coefficient. (**c**) A positive correlation was observed between p-GSK3β (S9) and KLF5 expression levels in the TNBC samples. Statistical significance was determined by the Spearman's test. (**d**) A positive correlation was observed between KLF5 and FGF-BP1 expression levels in the TNBC samples. Statistical significance was determined by the Spearman's test. (**e**) A positive correlation was observed between p-CREB (S133) and p-GSK3β (S9) expression levels in the TNBC samples. Statistical significance was determined by the Spearman's test. (**f**) The hypothetical model according to this study. When metformin is absent, accumulated cAMP activates PKA, which in turn phosphorylates GSK3β and causes GSK3β inactivation. KLF5 induces the transcription of its target genes, including *Nanog* and *FGF-BP1*. Metformin inhibits mitochondrial complex I, which results in a decrease in ATP and the accumulation of AMP. Accumulated AMP inhibits the generation of cAMP. Thus, PKA is inactive whereas GSK3β is active. Activated GSK3β directly phosphorylates KLF5 at S303, which leads to KLF5 degradation. TNBC stem cells could not be maintained without KLF5.

Since PKA stabilizes the KLF5 protein, we surmised that there might be a correlation between these two proteins. We first tested the expression of p-CREB, p-GSK3β, KLF5 and FGF-BP1 in a panel of breast cancer cell lines and found that p-CREB and p-GSK3β, KLF5 and FGF-BP1 are weakly or not expressed in non-TNBC cell lines compared with HCC1937 (Supplementary Figure S3). Following that, we performed immunohistochemical staining for KLF5 and p-CREB, which signify PKA activity, in human TNBC clinical samples. Remarkably, p-CREB positivity was observed in 72% (31 of 43) of TNBC samples (Figure 6a and b). At the same time, KLF5 was positive in 93% (40 of 43) of TNBC samples, which suggests that both activated PKA and KLF5 are upregulated in human TNBC (Figure 6b). Moreover, a strong positive correlation (*R*=0.541, *P*<1.8×10^−4^) between p-CREB and KLF5 was observed in these TNBC samples (Figure 6a and b). These data suggest that the overactivation of PKA may contribute to KLF5 overexpression in human TNBC. At the same time, we also observed positive correlations between KLF5 and p-GSK3β (*R*=0.385, *P*=0.017) (Figure 6a and c) and FGF-BP1 (*R*=0.412, *P*=0.008) (Figure 6a and d) in these TNBC samples. As expected, the expression of p-CREB and p-GSK3β was positively correlated (*R*=0.491, *P*=0.00176) (Figure 6a and e). These results suggest that the PKA-GSK3β-KLF5 pathway may be highly activated in TNBC patients.

## Discussion

Accumulating data suggest that metformin combined with other chemotherapeutics or radiotherapy may be used for the treatment of breast cancer. Metformin was shown to improve the response of cancer cells to chemotherapeutics or radiotherapy [22, 36]. Clinical data also showed that metformin improved the rate of pathologic complete response in diabetic patients with breast cancer who were treated with neoadjuvant chemotherapy [37]. However, negative results were also reported. In one small case study, metformin did not significantly promote the survival of diabetic patients with TNBC, although a trend has been noted that metformin decreased the risk of distant metastasis [26]. Therefore, the efficacy of metformin in TNBC requires further investigation.

Metformin has been reported to inhibit breast cancer stem cells [22]. However, the mechanism is not completely understood. In this study, we demonstrated that metformin significantly decreased the percentage of TNBC stem cells in two cell lines (Figure 1). Metformin inhibits the activity of PKA and subsequently activates GSK3β via a reduction in its phosphorylation. Activated GSK3β phosphorylates KLF5 at S303 and then targets it for ubiquitination and degradation (Figures 4, 5–6). In TNBC specimens, PKA is highly activated and is correlated with the expression of KLF5 (Figure 6). These results reveal a novel mechanism by which metformin significantly decreased the percentage of TNBC stem cells. It is well known that glucagon and epinephrine activate PKA [38, 39], and it is therefore possible that glucagon and epinephrine induce the expression of KLF5 protein.

KLF5 has a key role in metformin-induced TNBC stem cell reduction. Our previous results suggest that KLF5 is a stem cell transcription factor in basal-type TNBC cells [19]. KLF5 overexpression significantly rescued the TNBC stem cell reduction induced by metformin, which suggests that metformin acts, at least in part, through the inhibition of KLF5. It has been shown that metformin suppresses non-TNBC stem cells through nuclear factor-κB, signal transducers and activators 3 (Stat3) and mammalian target of rapamycin [40, 41]. Metformin was reported to induce apoptosis of TNBC cells through the inhibition of p-Stat3 [42]. However, we found that the knockdown of Stat3 did not decrease the KLF5 protein levels in TNBC cell lines (Supplementary Figure S4A and B).

Metformin inhibits mitochondrial complex I, which results in a decrease of ATP and the accumulation of AMP [32]. Accumulated AMP inhibits the generation of cAMP [32]. It has been established that cAMP activates PKA [32] and that activated PKA promotes mammary tumorigenesis [43]. Activated PKA also induces tamoxifen resistance in breast cancer [44]. We found that PKA has an important role in metformin-induced breast cancer stem cell suppression and that PKA is highly activated in triple-negative breast tumors. In agreement with our findings, metformin was reported to suppress breast cancer stem cells through the disruption of ATP production [45]. These results suggest that PKA is a potential target for the treatment of TNBC. In addition, energy stress such as glucose starvation has been demonstrated to increase the AMP level, and thus it is very likely that glucose starvation will also decrease the KLF5 protein level. Indeed, we observed that glucose starvation markedly decreased the KLF5 protein level (Supplementary Figure S5). It was reported that high glucose reduces the efficiency of metformin for breast cancer cell lines [46]. Indeed, metformin decreased the p-CREB, p-GSK3β and KLF5 levels at 5 mM glucose more efficiently than it did at 25 mM glucose (Supplementary Figure S5). Therefore, the induction of energy stress may benefit patients with KLF5-positive basal-like TNBC. Besides metformin, whether other PKA inhibitors, such as H89 [47], Rp-cAMPS [48] and PKIα [49] also inhibit the expression of KLF5 and TNBC needs further investigation.

In summary, we demonstrated that metformin significantly suppresses TNBC stem cells and revealed that metformin acts through the PKA-GSK3β-KLF5 pathway (Figure 6f). In addition, our results indicate that PKA and KLF5 are potential therapeutic targets in TNBC and support the idea that metformin is a promising agent for the treatment of patients with TNBC. These findings suggest that patients with high PKA activity and KLF5 expression may benefit more from therapies that contain metformin. Using this approach, in the future, we can design personalized therapies for patients with TNBC.

## Materials and Methods

### Cell culture

The human breast cancer cell lines HCC1806, HCC1937 and ZR-75-1 were cultured in RPMI-1640 medium supplemented with 10% fetal bovine serum, and MCF7 was cultured in MEM medium supplemented with 10% fetal bovine serum. T47D and SK-BR-3 were cultured in DMEM medium supplemented with 10% fetal bovine serum. BT474 was cultured in DMEM/F-12 (1:1) medium supplemented with 10% fetal bovine serum. All cells were maintained at 37 °C in an incubator with 5% CO_2_. The presence of mycoplasma was routinely tested by PCR to eliminate contamination.

### Drugs, reagents and antibodies

Metformin (D150959), 8-Br-cAMP (B7880), forskolin (F3917), cycloheximide, MG132, dimethyl sulfoxide and d-glucose were purchased from Sigma-Aldrich (St Louis, MO, USA). The anti-KLF5 and anti-p-KLF5 (S303) antibodies used for WB were described in our previous study [35]. The guinea-pig anti-klf5 antibody used for IHC was a gift from Pro. Huajing Wan of the Sichuan University (Chengdu, China). The anti-human FGF-BP1 (MAB1593) was purchased from R&D Systems (Minneapolis, MN, USA). The anti-HER2 (sc-284), anti-Nanog (sc-30331), anti-ER (sc-7207), anti-PR (sc-539) and anti-GAPDH (sc-25778) antibodies were purchased from Santa Cruz Biotechnology (Santa Cruz, CA, USA). The anti-β-actin (A5441) antibody was purchased from Sigma-Aldrich. The anti-p-CREB (S133) (9198), anti-CREB (9197), anti-p-GSK3β (S9) (9336), anti-GSK3β (9315), anti-p-Stat3 (9131) and anti-Stat3 (9132) antibodies were purchased from Cell Signaling Technology (Danvers, MA, USA).

### Flow cytometry

We performed an ALDH assay using an ALDEFLUOR Assay Kit (no. 01700; Stemcell Technologies, Vancouver, BC, Canada) according to the standard protocol. In brief, 20 000 cells were collected and resuspended in 1 ml assay buffer. Following that, 5 μl activated reagent was added. Half samples (0.5 ml) were immediately put into control tube that has 5 μl DEAB buffer. All samples were incubated for 45 min, and centrifuged for 5 min at 250 *g*. The cells were resuspended in 0.5 ml assay buffer and subjected to flow cytometry analysis.

### Mammosphere culture

We performed a mammosphere assay using a Mammosphere Culture Kit (no. 05620; Stemcell Technologies). HCC1806 and HCC1937 cells were digested into single cells and were plated in ultra-low attachment plates (no. 3473, Corning Inc., Corning, NY, USA) at a density of 500 or 3 000 cells per well. The cells were cultured in complete mammosphere culture medium (no. 05620; Stemcell Technologies). The number of mammospheres with a diameter >60 μm after 10–14 days in culture was then counted.

### Stable knockdown of KLF5

The KLF5 and control short hairpin RNAs were expressed in HCC1806 cell lines using the pSIH1-H1-puro lentiviral vector. Target sequences for KLF5 was 5′- CGAUUACCCUGGUUGCACA-3′ and control was 5′- CUUACGCUGAGUACUUCGA-3′. HCC1806 cells were selected using 1 μg ml^−1^ puromycin to generate stable cell populations.

### Tumorigenesis

HCC1806 cells (1×10^3^–10^6^) were suspended in 75 μl Matrigel (BD Biosciences, San Jose, CA, USA) and phosphate-buffered saline (PBS) at a 1:1 ratio and were injected into the fat pads of 7–8-week-old BALB/C nude mice from the Hunan SJA Laboratory Animal Co. Ltd (Changsha, Hunan, China). Tumor sizes were monitored from 7 days after injection. This animal experiment was approved by the animal ethics committee of the Kunming Institute of Zoology, CAS.

### Transfection

We used Lipofectamine 2000 (Invitrogen, Carlsbad, CA, USA) for siRNA and plasmid transfection according to the manufacturer’s recommended protocols. Control siRNA was purchased from RiboBio Co., Ltd (Guangzhou, Guangdong, China). The other siRNA sequences are listed in Supplementary Table S1. pBabe-KLF5 and pBabe empty vector have been described in our previous study[19].

### RT-qPCR

RNA was extracted using TRIzol reagent (Invitrogen). Reverse transcription was performed using the iScript cDNA Synthesis Kit (Bio-Rad) and RNA levels were quantified using SYBR Green Select Mastermix (no. 4472908, Applied Biosystems, Foster, CA, USA) on the ABI-7900HT System (Applied Biosystems). Primer sequences are listed in Supplementary Table S2.

### Immunohistochemical staining

Paraffin-embedded clinical TNBC specimens were obtained from the Kunming Medical University Affiliated Cancer Hospital (Kunming, China). The p-CREB, p-GSK3β (S9) and FGF-BP1 staining was performed according to the protocol described in our previous study [50]. The p-CREB (S133) antibody was diluted 1:800, p-GSK3β (S9) antibody was diluted 1:200 and FGF-BP1 antibody was diluted 1:100 for IHC. For KLF5 IHC, a guinea-pig anti-klf5 antibody was used and was diluted 1:2 000 in 4% goat blocking serum. The sections were incubated with the primary antibody overnight at 4 °C. Biotinylated anti-guinea-pig IgG (H+L) (no. BA-7000; Vector Laboratories Inc., Burlingame, CA, USA) was used as the secondary antibody, which was diluted 1:200 in 4% goat blocking serum. The sections were incubated with the secondary antibody for 30 min at room temperature on a shaker. Finally, the sections were incubated with avidin–biotin complex reagent for 30 min at room temperature on a shaker.

### Statistical analysis

Data are shown as means±s.d. Student’s *t*-test was used for statistical analysis, unless otherwise indicated. GraphPad Prism 6 (GraphPad Software Inc. La Jolla, CA, USA) and SPSS 20.0 Software (IBM Inc., Armonk, NY, USA) were used for all statistical analyses. *P*-values<0.05 were considered significant.
