# Supplementary material for: Metformin suppresses triple-negative breast cancer stem cells by targeting KLF5 for degradation
Source: Cell Discov. 2017 Apr 18;3:17010–. doi: 10.1038/celldisc.2017.10 (PMC5396048; doi:10.1038/celldisc.2017.10)
Supplement: Supplementary Information [file celldisc201710-s1.pdf]

Supplementary Table S1. siRNA sequences used in this study

Targets	Sequences (5' to 3')
PKAcα	CAAGGACAACUCAAACUUA
PKAcβ	AAGAGUUUCUAGCCAAAGCCA
GSK3β	AAGUAAUCCACCUCUGGCUAC
Stat3 1#	GGGACCUGGUGUGAAUUAU
Stat3 2#	CCCGGAAAUUUAACAUUCU
Stat3 3#	GGCCAGCAAAGAAUCACAU

Supplementary Table S2. qPCR primers used in this study

Names		Sequences (5' to 3')
KLF5	Forward	ACACCAGACCGCAGCTCCA
	Reverse	TCCATTGCTGCTGTCTGATTTGTAG
FGF-BP1	Forward	TGTTCAGAGGCTGTTTCCTG
	Reverse	TTCAGCAGAAAGTTCGTTGC
PKAcα	Forward	GATGCTGGTGAAACACAAGG
	Reverse	TCATTCAGGGTGTGTTCGAT
PKAcβ	Forward	GGGCTACAATAAGGCAGTGG
	Reverse	TGGTTGGTCTGCAAAGAATG
GAPDH	Forward	GGTGAAGGTCGGAGTCAACG
	Reverse	CAGAGTTAAAAGCAGCCCTGGT

Supplementary Figure Legend

Supplementary Figure S1. Metformin suppresses TNBC stem cells, as measured by the ALDH and mammosphere assay

A. The upper panel shows HCC1806 cells that were incubated with DEAB, a specific ALDH inhibitor. The lower panel shows the ALDH-positive CSCs. Metformin significantly decreased the percentage of ALDH-positive CSCs in HCC1806 cells. Representative results from at least three independent experiments are shown.

B. Metformin significantly decreased the percentage of ALDH-positive CSCs in HCC1937 cells.

C. Metformin inhibited ALDH-positive HCC1806 CSCs in a dose-dependent manner. The cells were treated with 2, 5, 10, 20 or 50 mM metformin for 24 h. The results are shown as bar graphs with the mean ± SD, n=3 independent experiments. The statistical significance was determined by student's *t-test*. **, $p<0.01$.

D. Representative pictures of mammosphere from HCC1806 and HCC1937.

Supplementary Figure S2. Overexpression KLF5 partially rescues the metformin-induced CSC reduction in HCC1937 cells, as measured by an ALDH assay. The upper panel shows HCC1937 cells that were incubated with DEAB, a specific ALDH inhibitor. The lower panel shows the ALDH-positive CSCs. Representative results from at least three independent experiments are shown.

Supplementary Figure S3. The PKA-GSK3β-KLF5 axis is not expressed in

non-TNBC breast cancer cell lines. ER-positive (ER+) cell lines: MCF7, T47D and ZR-75-1; HER2-positive (HER2+) cell lines: SK-BR-3 and BT474; and TNBC cell line: HCC1937 were collected. The protein expression levels were detected by WB.

Supplementary Figure S4. Stat3 knockdown does not downregulate the expression levels of KLF5 in TNBC cell lines. Three different Stat3 siRNAs efficiently silenced Stat3 expression in HCC1806 and HCC1937 cells; however, the KLF5 protein levels were unchanged. Cells were transfected with siRNAs for 72 h. Stat3, p-Stat3 (Y705) and KLF5 levels were measured by WB.

Supplementary Figure S 5. Glucose starvation decreases the expression of KLF5 and increases the efficacy of metformin in TNBC

A. HCC1937 cells were cultured in glucose (25 mM) or glucose-free medium for 24 h. The WB result was shown.

B. HCC1937 cells were cultured for 24 h and were washed at least 3 times by PBS. Following that, the cells were cultured in glucose-25 mM or glucose-5 mM medium. Metformin was added for 24 h and the cells were collected for WB.

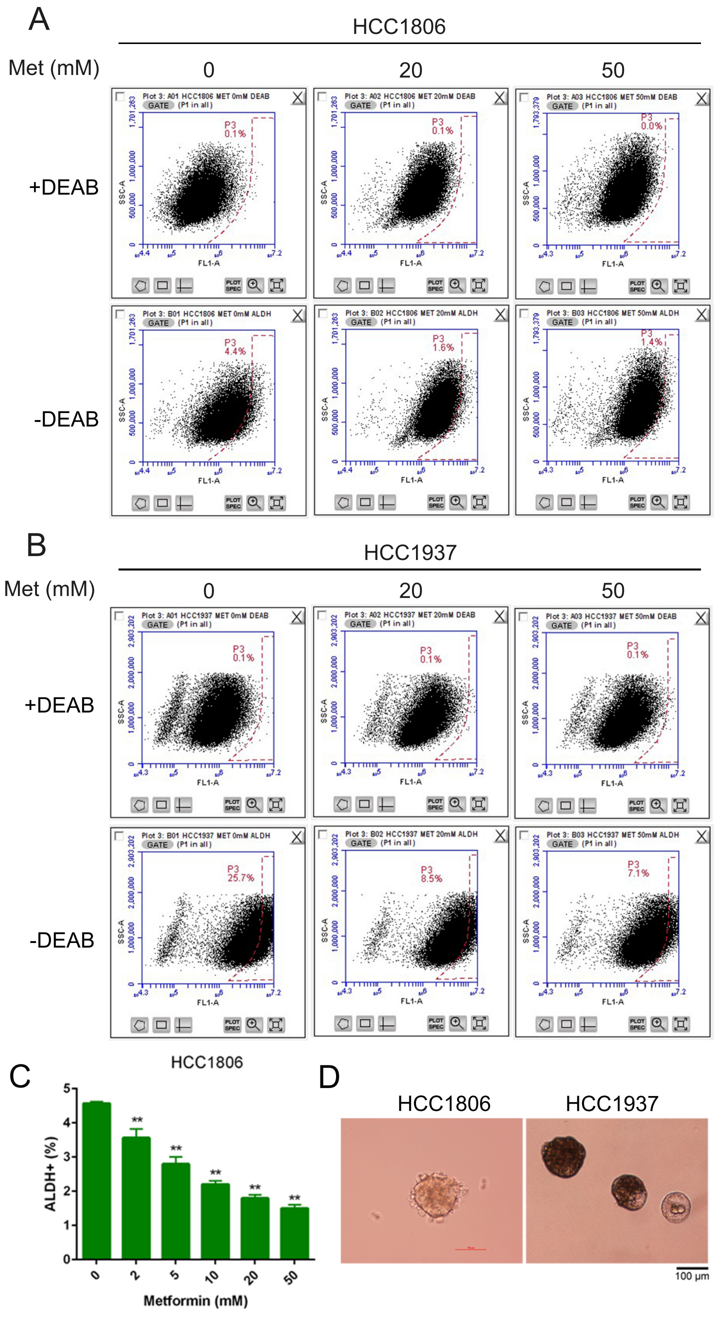

Supplementary Figure S1

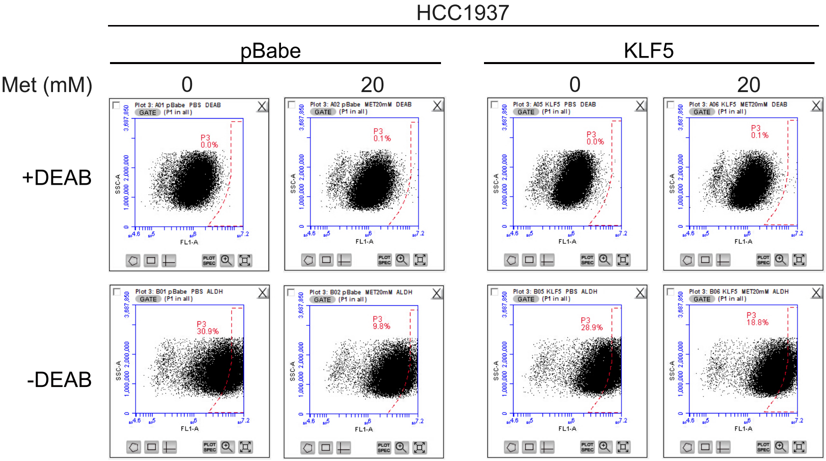

Supplementary Figure S2

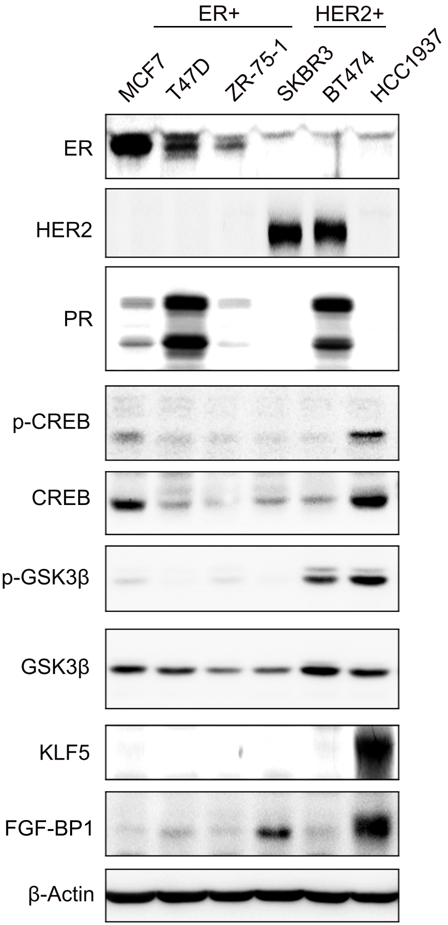

Supplementary Figure S3

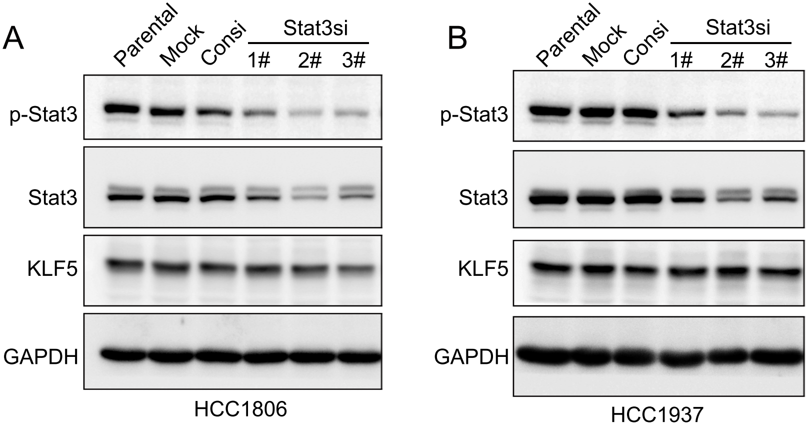

Supplementary Figure S4

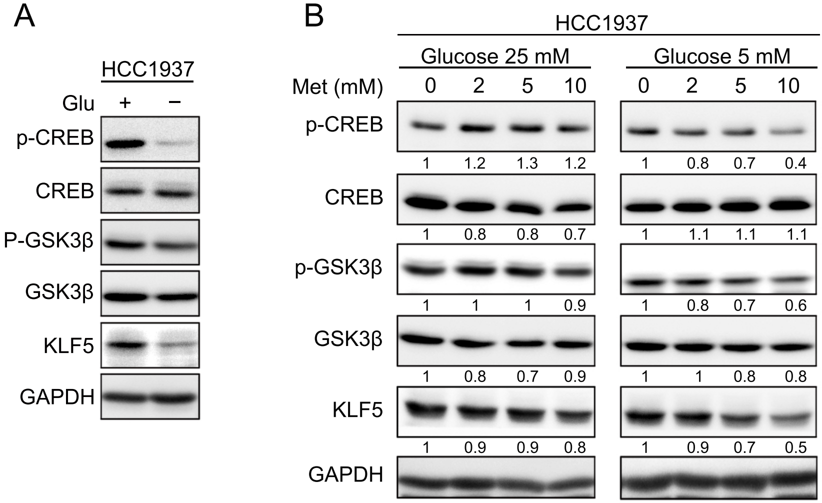

Supplementary Figure S5
